# Impact of *Schistosoma mansoni* Infection on the Gut Microbiome and Hepatitis B Vaccine Immune Response in Fishing Communities of Lake Victoria, Uganda

**DOI:** 10.3390/vaccines13040375

**Published:** 2025-03-31

**Authors:** Yan Wang, Ariana K. Waters, Geofrey Basalirwa, Ali Ssetaala, Juliet Mpendo, Annemarie Namuniina, Emily Keneema, David Kiiza, Jacqueline Kyosiimire-Lugemwa, Yunia Mayanja, Brenda Okech, Sylvia Kiwuwa-Muyingo

**Affiliations:** 1 Public and Population Health, School of Dentistry, University of California, Los Angeles, CA 90095, USA; 2 School of Public Health, University of California, Berkeley, CA 94704, USA; 3 UVRI-IAVI HIV Vaccine Program, Entebbe P.O. Box 49, Uganda; 4 Medical Research Council/Uganda Virus Research Institute and London School of Hygiene and Tropical Medicine (MRC/UVRI & LSHTM) Uganda Research Unit, P.O. Box 49, Plot 51-59 Nakiwogo Road, Entebbe 256, Uganda; 5 University of Kisubi, Faculty of Health Science and Department of Biomedical Sciences, Entebbe P.O. Box 182, Uganda; 6 African Population & Health Research Center (APHRC), Nairobi Kenya P.O. Box 10787-00100, Kenya

**Keywords:** HBV vaccine, microbiome, *S. mansoni* infection

## Abstract

Objective: *Schistosoma mansoni* (*S. mansoni*) infection is endemic in Ugandan fishing communities. We investigated its potential impact on Hepatitis B (Hep B) vaccine responses and its role in mediating the association between the gut microbiome and long-term effectiveness of the vaccine. Methods: Participants were tested for *S. mansoni* infections at baseline and received the Hep B vaccine at baseline, month 1, and month 6. Those with infections were treated. Stool samples were collected at baseline and analyzed using 16S rRNA sequencing. The Wilcoxon rank-sum test was used to compare alpha diversity between groups. A linear regression model was applied to estimate the association between one-year Hep B vaccine responses and the baseline gut microbiome by infection status, adjusting for age and sex. Results: A total of 107 participants were included (44 from the fishing community and 63 from the Kampala community). There was no significant difference in microbiome composition by location or infection status at baseline or discharge. In the linear regression analysis, *S. mansoni* infection (β = 1.24, *p* = 0.025) and a higher alpha diversity (β = 0.001, *p* = 0.07) were associated with higher Hep B vaccine responses, while older age was associated with a lower Hep B vaccine response (β = −0.06, *p* = 0.0013). Conclusions: *S. mansoni* infection status before vaccination may modify the association between the gut microbiome and Hep B vaccine response. Potential interventions could focus on infection control as well as improving microbiome richness before implementing vaccine programs in fishing communities.

## 1. Introduction

Vaccine efficacy remains an area of concern in vaccine science. Increasing evidence has shown that the microbiome modulates immune response [1,2,3]. The co-development of the immune system and microbiome occurs during the first two years of life [1,4,5]. A lack of microbial stimulation will lead to an underdeveloped immune system, as microbial stimuli drive key processes in the maturation of adaptive immune cells [6,7]. This interplay between gut microbiota and the immune system may partially account for the variability in vaccine response [8,9,10,11]. In humans, specific bacterial taxa have been linked to variations in vaccine efficacy [10,11,12]. However, few studies have investigated the influence of the gut microbiome on vaccine response, especially among adults [3]. For example, *Actinobacteria* were positively associated with adaptive immune responses to systemic vaccines like the bacille Calmette–Guérin (BCG) vaccine, tetanus toxoid (TT) vaccine, Hepatitis B virus (HBV) vaccine, and oral polio vaccine (OPV) in a study on Bangladeshi infants. Conversely, the bacterial diversity and abundance of *Enterobacteriales*, *Pseudomonadales*, and *Clostridiales* were associated with lower vaccine response, measured by vaccine-specific T-cell proliferation [13].

The co-development of the gut microbiota and immune system makes it a beneficial mutualistic relationship, allowing the host to maintain a balance between active immunity and pathogens and vaccines [2,14]. Probiotics with specific strains have been shown to modulate the gut microbiome and systemic immune response, and they provide a basis for studies on vaccines in humans [15,16,17]. Oral treatment with *Clostridium* bacteria was shown to promote the expansion of regulatory T-cells (Tregs) in the intestines of early-life mice, conferring resistance to colitis and augmented immune response in adulthood [18]. A study using germ-free or antibiotic-treated mice demonstrated impaired antibody responses to influenza vaccines, which were restored through the oral dosing of a flagellated strain of *E. coli* [19]. The use of microbiome modulation to boost vaccine response in humans has yielded mixed results in previous clinical trials, but it shows potential for further study [14,20,21,22].

In low- and middle-income countries, intestinal dysbiosis is associated with environmental influences that can negatively impact vaccine efficacy, such as commonly occurring undernourishment and gastrointestinal infections [2]. Schistosomiasis is a neglected tropical disease caused by infection with *Schistosoma* flatworms. *Schistosoma mansoni* (*S. mansoni*) infection is endemic in Uganda, with a national prevalence of 25.6% [23]. Fishing communities have a particularly high burden and socio-economic vulnerability to *S. mansoni* infection, with approximately 51–57% of the population being infected despite door-to-door treatment campaigns [24,25,26,27]. Treatment consists of a single oral dose of Praziquantel (PZQ). *S. mansoni* infections impact the immune system and possibly enhance the virulence of hepatrophic viruses [28]. While about half of *schistosoma* eggs are carried through the bloodstream to the liver, the other half attach to the endothelium and migrate through the intestinal tissues. These eggs release a mixture of enzymes and antigens, which promote the recruitment of immune cells, the development of inflammatory infiltrates, and the formation of tissue granulomas at the site of egg deposition within the intestinal wall [29]. Multiple studies have shown that the resulting inflammation results in varied changes in the alpha and beta diversity of the host microbiome compared to uninfected controls, suggesting that there is likely a complex interplay between schistosomes, the gut microbiota, and the inflamed host immune system [30].

Hepatitis B (Hep B), a life-threatening disease caused by HBV, is highly endemic in Uganda, with a national prevalence of approximately 4.3%. The burden is substantially higher among fishing communities (7%) compared to the global prevalence of 3.9% [31]. Transmission occurs through mucosal or percutaneous exposure to infected blood or other body fluids [32]. Over 80% of individuals with chronic HBV are unaware of their status, preventing them from accessing care and treatment interventions aimed at reducing transmission [33]. Consequently, vaccination remains the most effective strategy for protecting populations against HBV. An estimated 52% of Ugandan adults remain infected with HBV despite the incorporation of the Hep B vaccine into early childhood immunization programs [32]. Although the Hep B vaccine is effective, 5–10% of individuals do not develop an immune response [34,35]. It has also been shown that Hep B-infected individuals experience compositional changes in their gut microbiota relative to healthy controls [36,37].

The risk of both Hep B and *S. mansoni* infection is high in Lake Victoria fishing communities [31]. To better understand the relationship between *S. mansoni* infection, the gut microbiome, and Hep B vaccination, we analyzed samples from a cohort of fisherfolk participants in Uganda who were part of a simulated vaccine efficacy trial (SiVET). In this trial, the ENGERIX-B Hep B vaccine, a well-established vaccine against Hepatitis B, was administered to Hep B antigen-negative individuals with varying levels of exposure to *Schistosoma.* At the conclusion of the SiVET trial, we conducted a follow-up study to characterize the microbiomes of PZQ-treated individuals at pre- and post-immunization time points, focusing on those with high and low serum worm burdens. We hypothesized that alterations to the baseline composition of the microbiome due to *S. mansoni* infection would be associated with long-term Hep B vaccine efficacy. This work aimed to investigate a possible link between *S.mansoni* infection and the microbiome-related immune response to Hep B immunization in order to inform vaccination practices among impacted communities in low-income countries.

## 2. Materials and Methods

### 2.1. Study Design

**Figure 1:**
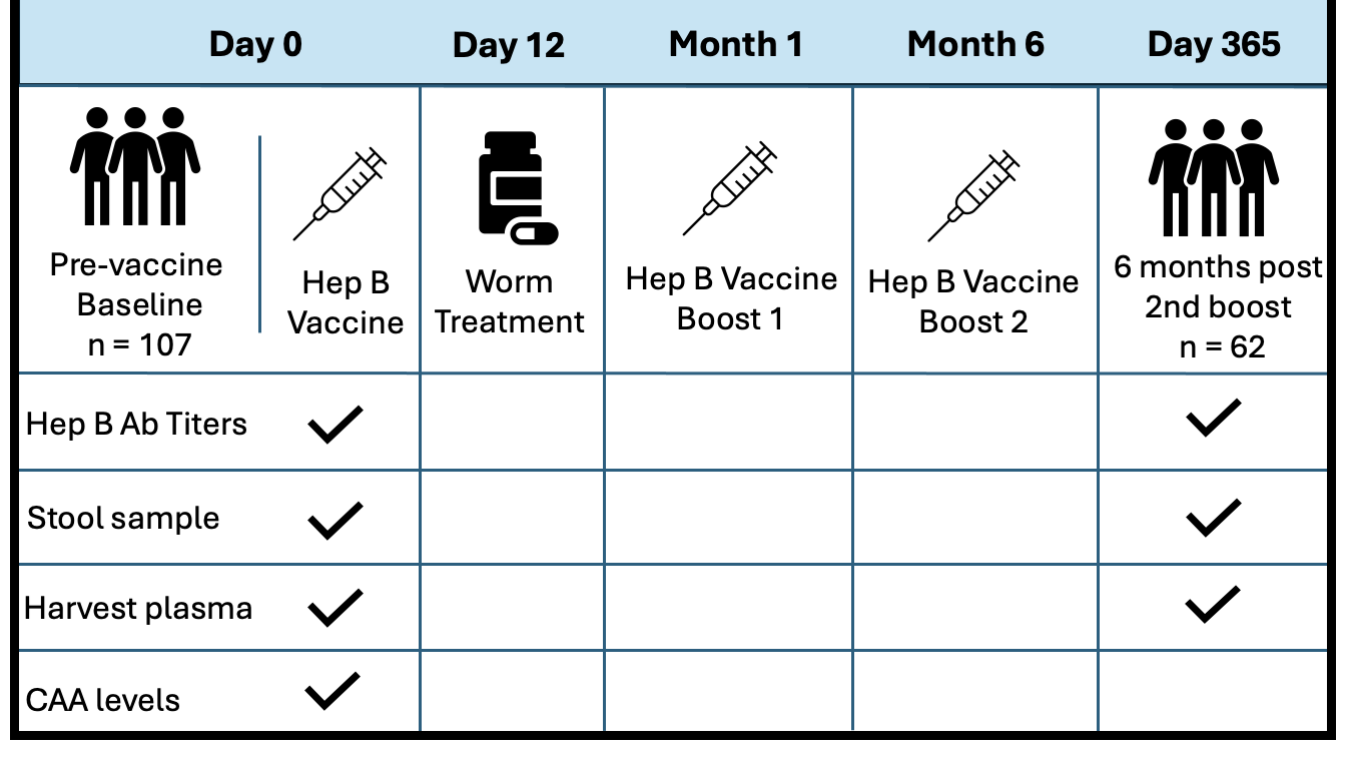
Study design and sample collection schedule. Sample collection is indicated with a checkmark.

This was a prospective observational study, where participants were enrolled as part of a larger community-based simulated vaccine efficacy trial (SiVET) in Uganda. For this study, healthy adult males and non-pregnant females aged 18–49 were enrolled from two communities: a highly *S. mansoni*-burdened fishing community (Kigungu) [25] and a low-*S. mansoni*-burden cohort from the Good Health for Women Project clinic located in Kampala [38]. To prevent potential confounding from previous Hepatitis B exposure, all study participants were required to provide negative Hep B surface antigen and core antibody tests. The other inclusion criteria for this study included an ability to participate and give written informed consent for the Hep B vaccine, a willingness to consent for 12-month follow-up after the first immunization, and that they be HIV-uninfected. The first dose of the Hepatitis B vaccine, ENGERIX-B (GlaxoSmithKline Biologicals), was administered within 6 weeks of pre-screening. Booster doses were provided one month and six months after the initial injection. Doses were injected intramuscularly into the deltoid muscle at a concentration of 20 ug/mL. Stool samples were collected at baseline and six months post all doses (one-year follow-up). *S. mansoni* infection status was determined by inspection of participant stool samples for the presence of *schistosoma* eggs. If stool samples were considered positive, patients were treated with a single dose of 40 mg/kg body weight (average 2.4 g) of praziquantel (PZQ) on D12, following the first dose of the Hep B vaccine. Figure 1 illustrates the study design at baseline and discharge. PBMCs and plasma were collected before vaccination at D0 and after one year of follow-up (D365). Patient plasma was evaluated for serum schistosome-specific circulating anodic antigen (CAA), a quantitative biomarker of *S. mansoni* infection.

### 2.2. Circulating Anodic Antigen (CAA) Assay

Worm burden was quantified using a circulating anodic antigen (CAA) assay, which measures *Schistosoma*-derived antigens in the bloodstream. CAA levels serve as a direct indicator of living worm presence, reflecting ongoing infection and associated worm burden. Analysis to determine active *S. mansoni* infection was carried out on Day 0, prior to the first vaccination, using frozen serum samples and techniques described previously [25]. A calibration curve for establishing CAA cutoff levels and sample quantification was created using CAA concentration standards and negative serum controls. Participant samples were then evaluated using either the SCAA500 diagnostic method with a detection limit of 3 pg/mL or the SCAA20 test format with a 30 pg/mL detection threshold for low-volume samples. CAA lateral flow strips were incubated overnight in wells containing 20 µL of concentrated patient sample and analyzed the following day using an Upcon plate reader (Labrox Oy, Turku, Finland). The flow control signals (FC) of the individual strips were used to normalize the test line signals (T; relative fluorescent units, peak area), and the results were expressed as a ratio value (R = T/FC).

### 2.3. Hepatitis B Antibody Testing

Serum samples were assayed for three markers of HBV infection during study screening to confirm a Hep B-negative status prior to vaccination and to differentiate active infection from past exposures. Hep B surface antibody (anti-HBs) testing was performed using the Cobas e 411 analyzer (Roche Diagnostics, Mannheim, Germany), using a cutoff value of 10 IU/L. Hep B core antibodies (anti-HBc) were tested for using the VIDAS anti-HBc Total II kit (BioMérieux SA, Marcy-l’Étoile, France), and Hepatitis B surface antigen (HbsAg) was tested for using the VIDAS HbsAg Ultra kit (BioMérieux SA, Marcy-l’Étoile, France). Both kits were run on a MinVidas analyzer (BioMérieux SA, Marcy-l’Étoile, France). During subsequent follow-up visits, only the anti-HBs antibody was assayed.

### 2.4. Microbiome Analysis Using 16S rRNA Sequencing

Microbial DNA was extracted from participant stool samples. The V4 hypervariable regions of the 16S rRNA gene were amplified using barcoded universal primers and PCR. Amplified DNA was purified, quantified, and pooled using the 300 bp paired-end protocol, as described previously [39]. Samples were then sequenced on the Illumina MiSeq platform. Read quality from sequencing was assessed using a standardized bioinformatics pipeline from the NIH Human Microbiome Project’s standard operating procedures [40,41,42]. Operational taxonomic units (OTUs) were clustered according to the Greengenes database [43,44]. Taxonomic classification was conducted using UTAX via USEARCH [45], a high-throughput sequence analysis tool integrated into QIIME [43,44,46], an open-source microbiome bioinformatics platform. Jensen–Shannon distances between pairs of community states were computed for each sample and grouped hierarchically using Ward linkage to cluster vectors of phylotype proportions into community state types [47].

### 2.5. Statistical Analysis

All statistical analyses were performed using R Statistical Software (v4.4.1), and the R packages phyloseq (v1.48.0) and microbial (v0.0.21). Four alpha diversity metrics were used to evaluate the gut microbiome: Chao1 and the abundance-based coverage estimator (ACE), both of which measure community richness, and the inverse Simpson and Shannon indices, which measure community diversity. Wilcoxon rank-sum tests were used for the statistical evaluation of alpha diversity between non-*S. mansoni*-infected (NI) vs. infected individuals with CAA levels greater than 3 pg/mL. Beta diversity distance was visualized using Bray–Curtis Principal Coordinate Analysis (PCoA). The linear regression model’s outcome variable was Hep B effectiveness vs. Chao1 alpha diversity, adjusted for infection status at baseline. Infection status was a mediating factor between microbiome diversity and the long-term effectiveness of the vaccine. Bivariate analyses were used to test the difference in alpha diversity by infection status and by location. Linear regression analyses were adjusted for age and sex, and paired t-tests were used to evaluate the significance of the association. 

### 2.6. Ethical Approval

This study received ethical approval from the Uganda Virus Research Institute Research Ethics Committee, reference number GC/127/15/07/439, and the Uganda National Council of Science and Technology, reference number HS 1850, approved on 2 July 2016. Documented written informed consent was obtained from all participants before taking part in any study procedures.

## 3. Results

We recruited a total of 113 study participants. Six participants were excluded due to having an unknown or unclear infection status. The final analysis included 107 participants recruited at baseline, with 63 (59%) from the Kampala site and 44 (41%) from the fishing community (see Table 1). There was no significant difference in age by site or infection status (*p* = 0.261). Over half of the participants [58%, 62/107] completed the three-dose Hepatitis B vaccine treatment, with all samples available, including plasma and stool samples for gut microbiome analysis. The mean age of the two communities was 28.1 years, with the majority being female (70%). Approximately 42 individuals (39%) had *S. mansoni* infection at baseline. There was a significant difference by sex (*p* < 0.001) and infection status (*p* < 0.001) at baseline between the two sites. These two variables were adjusted for as covariates in the regression model.

### 3.1. Microbiome Composition Analysis

**Figure 2:**
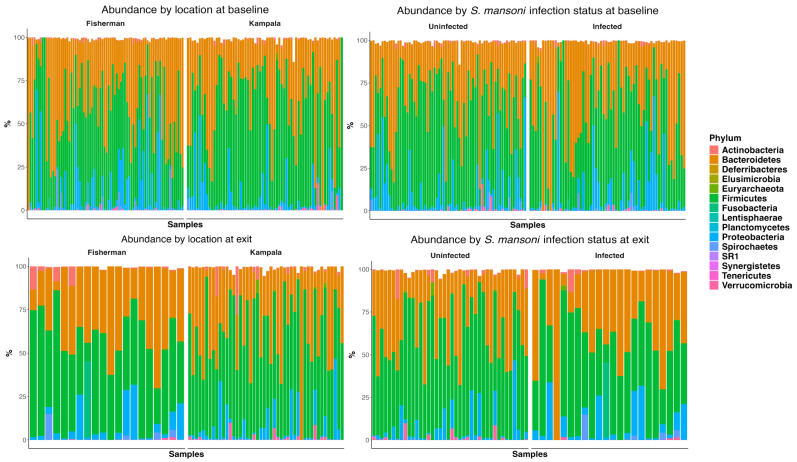
Abundance plot at phylum level by site, by infection status at baseline, and at one-year follow-up.

Figure 2 depicts the abundance plots at the phylum level by location and infection status at both baseline and D365. There was no significant difference in microbiome composition at the phylum level. Firmicutes and Bacteroidetes were the primary phyla present for all study groups, followed by proteobacteria. Welch’s two-sample t-test indicated no significant difference between infection statuses at baseline in terms of the Shannon (*p* = 0.24), Chao 1 (*p* = 0.30), ACE (*p* = 0.16), and inverse Simpson (*p* = 0.12) indices. Similarly, there was no significant difference at discharge regarding the Shannon (*p* = 0.07), Chao 1 (*p* = 0.98), ACE (*p* = 0.99), and inverse Simpson (*p* = 0.08) indices.

**Figure 3:**
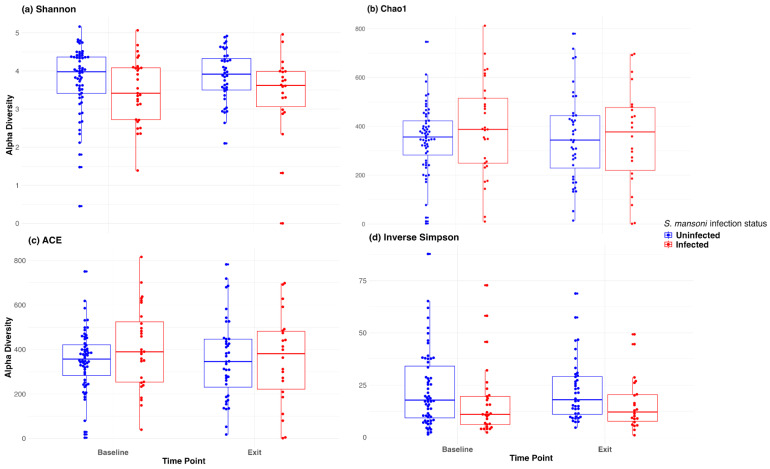
Box plot of alpha diversity (Shannon, Chao1, ACE, and inverse Simpson) at baseline and at discharge (month 12).

**Figure 4:**
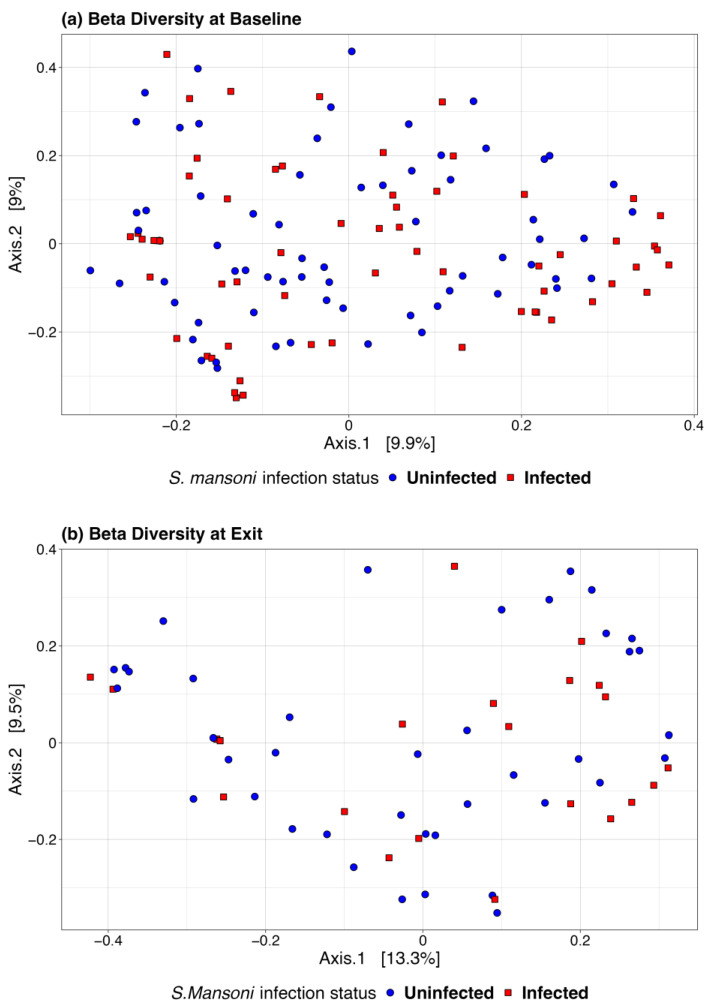
Bray–Curtis beta diversity at baseline (**a**) and discharge (**b**) by infection status.

We also used a paired *t*-test to determine whether there was a significant change in the microbiome between baseline and 12-month exit. No significant differences were observed by infection status using any of the above alpha diversity measures. Figure 3 includes a box plot comparing alpha diversity measures by infection status over time. None of the comparisons indicated significance, as all *p*-values were greater than 0.05. The microbiome at baseline and exit were relatively stable for the participants at both sites and by infection status. Figure 4 presents the Bray–Curtis measure for beta diversity by infection status at baseline and at discharge. We did not observe significant differences or patterns of beta diversity by infection status. At both baseline and discharge, there was no clear separation or diversity pattern by infection status for all measures, indicating that the microbial communities are not significantly different.

### 3.2. Regression Model

**Figure 5:**
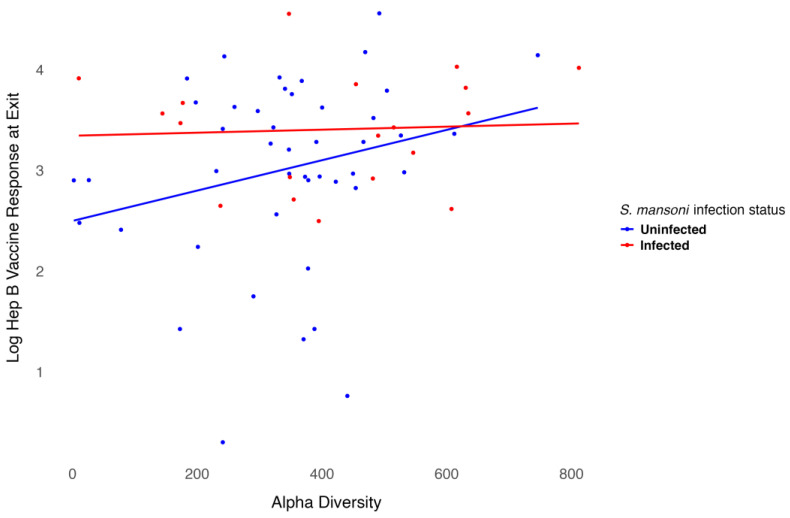
Relationship between Hep B level and alpha diversity at baseline by infection status.

We modeled the log-transformed Hep B vaccine antibody level at discharge, which was six months after the three-dose vaccination series, including baseline alpha diversity, infection status, and their interaction, while adjusting for age and sex. Age was negatively associated with the six-month antibody level after vaccination (*p* = 0.001), as shown in Table 2. Females exhibited higher antibody levels compared to males, though this association was not significant. However, infection status was significantly associated with antibody levels (*p* = 0.02). For participants infected with *S. mansoni*, baseline alpha diversity measures were associated with lower six-month antibody levels after vaccination, even though treatment was provided before vaccination. In contrast, for participants who were not infected, baseline alpha diversity measures were associated with higher six-month antibody levels after vaccination. The interaction between infection status and alpha diversity was not significant (Figure 5). Overall, baseline alpha diversity, infection status, their interaction, age, and sex accounted for 25% of the variation in antibody levels six months after the three-dose Hep B vaccination series. Included in the supplementary materials are plots of the regression analysis using other alpha diversity measures. Though the trend remains somewhat similar, infection status is not significant using the Shannon (Figure S1), ACE (Figure S2), or inverse Simpson (Figure S3) indices.

## 4. Discussion

In this study, we evaluated the role of the microbiome and *S. mansoni* infection status in the response and effectiveness of the Hep B vaccine. We worked with two infections simultaneously: *S. mansoni* infection (treated) and the immune response to the Hep B vaccine, measured by antibody levels. No significant differences in the microbiome composition were observed at any given time point or location. Our model suggests that *S. mansoni* infection status and age are important for the long-term effectiveness of the intravenous Hep B vaccine. Though our study did find an interaction between Chao1 alpha diversity and Hep B antibody level that differed by infection status, the results were not statistically significant (*p* = 0.08). The chao1 index accounts for rare or underrepresented species that might otherwise be missed in samples with a lower sequencing depth. Given this and the borderline significance of our results, the baseline microbiome may have still played a role in the long-term effectiveness of the Hep B vaccine, differing by *S. mansoni* infection status even when treatment was provided before vaccination. Detecting this relationship more definitively may require deeper and more robust sequencing. 

Our results are in line with growing evidence linking the intestinal microbiome to immune function and vaccine efficacy, but the mechanism of this linkage remains unclear. Existing research highlights a complex, bidirectional relationship; for example, a longitudinal cohort study of COVID-19 vaccines found that *F. prausnitzii* was associated with strong and sustained antibody responses following mRNA vaccination, while *E. coli* was linked to slower antibody decay after adenoviral vaccination [48]. Similarly, a randomized clinical trial on the MucoRice-CTB cholera vaccine found that those who responded to vaccination exhibited increased gut alpha diversity compared to non-responders and that neutralizing antibodies against diarrheal toxins were generated in a microbiota-dependent manner [49]. In contrast, a separate study of cholera vaccination in Bangladesh found no direct correlation between gut diversity and most immune responses, though individuals with higher levels of *Clostridiales* and lower levels of *Enterobacterales* were more likely to develop a memory B cell response [50]. These and other findings suggest that while the gut microbiota can influence vaccine-induced immune responses and vaccination itself can alter the microbiome, identifying the specific microbiome features that correlate with vaccine effectiveness remains a key challenge [7]. The fundamentally dynamic nature of the human microbiome and geographic differences in baseline composition and diversity complicate these studies, and more work is needed to make causal connections [51,52].

Regardless of the role of the microbiome, our results clearly identified that *S. mansoni* infection status impacts long-term Hep B vaccine efficacy. Chronic helminth infections have previously been associated with a hyporesponsive immune system and have been hypothesized to contribute to reduced vaccine efficacy in sub-tropical regions [53]. A potential mechanism of *S. mansoni* immune modulation is the elevation of T-regulatory cells and the suppression of T-helper cells and adaptive immune response, which can improve after treatment with praziquantel [54,55]. A recent study investigating measles immunization and schistosomiasis among Ugandan pre-school children, which found that *S. mansoni* infection was associated with an impaired immune response that improved with praziquantel treatment [56], is consistent with our results.

In this study, *S. mansoni* infection was treated with praziquantel (PZQ). Patients from villages or those with previous infections may occasionally need higher doses due to PZQ resistance, which can develop among *S. mansoni* populations. Although PZQ resistance is relatively low among Ugandan fishing communities, intensive PZQ use in these areas due to the recurrence of the infection necessitates an alternative drug to treat *S. mansoni*, as already noted in the literature [57,58,59]. This study provides evidence that treating *S. mansoni* infection before immunization may be beneficial, as infection status may have a lasting impact on the effectiveness of vaccination. We did not evaluate the biological mechanisms underlying how certain infection statuses might interact with the immune system through microorganisms that influence antibody-level development after vaccination. Further investigation is needed to explore the effects of infections, such as *S. mansoni*, on immune response and systemic health.

## 5. Limitations

This study has several limitations. These included significant loss during follow-up, the limited availability of true negative controls (i.e., unexposed individuals living in parasite-endemic fishing villages), and a lack of data on potential lifestyle confounders (i.e., diet, smoking, alcohol use). Participants were only screened for HIV, so there is still a risk of confounding due to co-infection with other common diseases such as malaria or tuberculosis.

The fishing community had high mobility during the one-year follow-up of the study [60,61], with most people moving out due to economic challenges, especially among females [62,63,64]. Despite the limited sample size, we were still able to demonstrate the necessity of infection control and the importance of maintaining a balanced gut microbiome, which can benefit long-term vaccine effectiveness. *S. mansoni* infection is very common in the fishing community, with infection and reinfection occurring frequently. It was almost impossible to find individuals who were not infected with *S. mansoni* at the time of Hepatitis B vaccine initiation in this area. The purpose of this study was to evaluate the association of this infection with, as well as its impact on, the microbiome and the long-term effectiveness of the vaccine. We collected lifestyle variables such as diet, alcohol use, and other factors that may impact the long-term effectiveness of the vaccine. However, we did not include them in this analysis due to inconsistencies and to avoid a further reduction in sample size and statistical power. We aim to emphasize a global-level analysis and an overall evaluation of the importance of future interventions targeting *S. mansoni* infection in this community.

The Kampala cohort (Good Health for Women cohort), set up between 2008 and 2009, was established to recruit women involved in high-risk sexual behavior, resulting in a gender imbalance by design [65]. The aim was to better understand the dynamics of HIV/STI infection to inform future HIV prevention intervention trials in this group. Studies have documented high rates of HIV and STIs among women involved in high-risk behaviors, and the prevalence of STIs in the cohort, including HIV, is high (37%). As HBV is transmitted both sexually and non-sexually, it is plausible that this cohort of high-risk women may not account for a wide range of exposure risks that are present in fishing communities [66,67,68]. In both fishing communities and high-risk cohorts of women, sexual transmission is a potential dominant route of transmission for HBV [69]. The findings here should be interpreted considering variations in sex distribution and background characteristics.

The Chao1 index indicated differences in rare and underrepresented species, but our sequencing depth was insufficient to evaluate species-level variations, as 16S data are only reliable up to the genus level. This index is an overall alpha diversity measure based on the abundance of singletons (species found only once) and doubletons (species found twice) in a sample [70,71]. Given the limited sample size in this study and critiques in the literature of the Chao1 index regarding low-abundance classes, we did not analyze this aspect in detail to avoid misleading results. The findings here suggest that future large-scale studies should investigate rare species that may impact the effectiveness of the vaccine. Nevertheless, this study provides a foundation suggesting that certain rare species may influence vaccine effectiveness in the long term. Longer and more in-depth studies are needed to investigate the role of species-level variations on vaccine effectiveness, particularly under different infection statuses. Additionally, this study evaluated a population in Uganda, where Hep B infection and *S. mansoni* infection are highly prevalent.

## 6. Conclusions

This study evaluated a population in Uganda, where Hepatitis B and S. *mansoni* infections are highly prevalent. We suggest that vaccine programs should consider addressing infection status and microbiome richness before implementing longer-dose vaccination schedules. In this study, we provided treatment for *S. mansoni* infection before initiating the Hep B vaccination, adhering to ethical regulations. This approach underscores the importance of conducting health and wellness checks prior to immunization. The complex interplay between the microbiome and vaccine efficacy could be influenced by parasitic infection status. While potential environmental, social, and behavioral factors were not considered, these findings provide valuable insights for future studies. For example, dietary interventions to optimize baseline microbiome status and the treatment of infections, particularly in areas with a high prevalence of infections and malnutrition, may improve the effectiveness of Hep B vaccine antibody levels. This study provides a foundation, suggesting that certain rare species of bacteria may influence vaccine effectiveness in the long term. Longer and more in-depth studies are needed to investigate the role of species-level variations in vaccine effectiveness, particularly under different infection statuses.

## Figures and Tables

**Table 1:** Characteristics of the participants from Kampala site and Fishing Community site.

Baseline Characteristic	Full Sample	Kampala	Fisherman	
	*n* (%)	*n* (%)	*n* (%)	*p*-Value ^1^
Age Category (years)				
15–25	39 (36)	24 (38)	15 (34)	0.261
26–35	51 (48)	27 (43)	24 (55)	
36+	16 (15)	12 (19)	4 (9)	
No age given	1 (1)	0	1 (2)	
Mean age (SD)	28.1 (5.9)	28.0 (6.3)	28.2 (5.3)	
Gender				<0.001
Female	75 (70)	63 (100)	12 (27)	
Male	31 (29)	0	31 (71)	
Not given	1(1)	0	1 (2)	
*S. mansoni* Infection				<0.001
Yes	42 (39)	8 (13)	34 (77)	
No	65 (61)	55 (87)	10 (23)	
Total	107	63(59)	44 (41)	

^1^ Chi-Square *p*-value.

**Table 2:** Regression analysis of Hep B vaccine antibody levels.

Effect	Estimate	SE	95% CI		*p*
			LL	UL	
Intercept	3.773	0.629	2.514	5.031	0
Chao1	0.001	8 × 10^−4^	−0.000	0.003	0.076
Infected (yes)	1.241	0.538	0.164	2.317	0.025
Age (years)	−0.057	0.017	−0.091	−0.023	0.001
Sex (female)	0.389	0.313	−0.237	1.015	0.218
Chao1 × Infection status	−0.002	0.001	−0.004	0.0004	0.113
Multiple R^2^	0.25				
Adjusted R^2^	0.19				

Note. SE = standard error; CI = confidence interval; LL = lower limit; UL = upper limit.

## Data Availability

All data supporting this study are available upon reasonable request to the corresponding authors.

## Supplementary Materials

The following supporting information can be downloaded at https://www.mdpi.com/article/10.3390/vaccines13040375/s1, Figure S1: Relationship between Hep B level and alpha diversity at baseline by infection status, Shannon Index; Figure S2: Relationship between Hep B level and alpha diversity at baseline by infection status, ACE Index; Figure S3: Relationship between Hep B level and alpha diversity at baseline by infection status, inverse Simpson
