# Supplementary material for: Impact of Schistosoma mansoni Infection on the Gut Microbiome and Hepatitis B Vaccine Immune Response in Fishing Communities of Lake Victoria, Uganda
Source: Vaccines (Basel). 2025 Mar 31;13(4):375. doi: 10.3390/vaccines13040375 (PMC12030974; doi:10.3390/vaccines13040375)

Supplementary

Regression analysis comparing alpha diversity to Hepatitis B vaccine titer level at study exit, by infection status.

S1. Shannon index

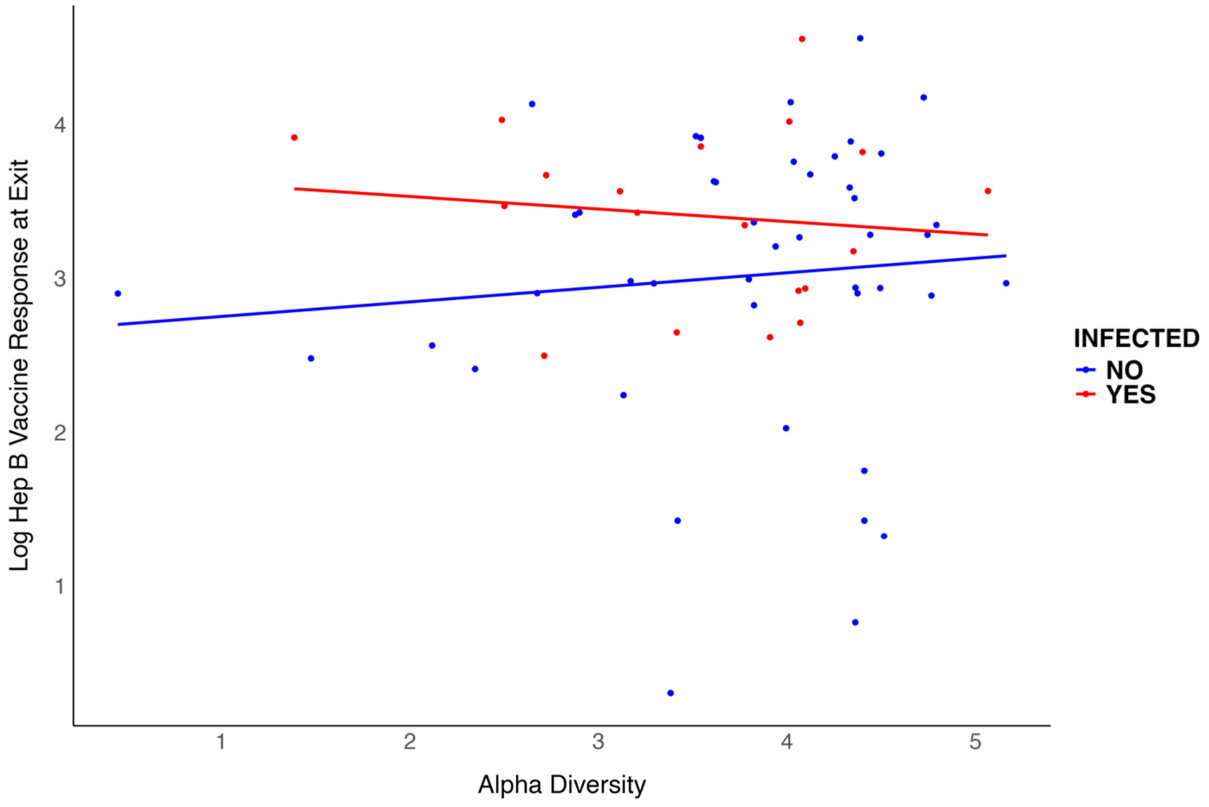

S2. ACE index

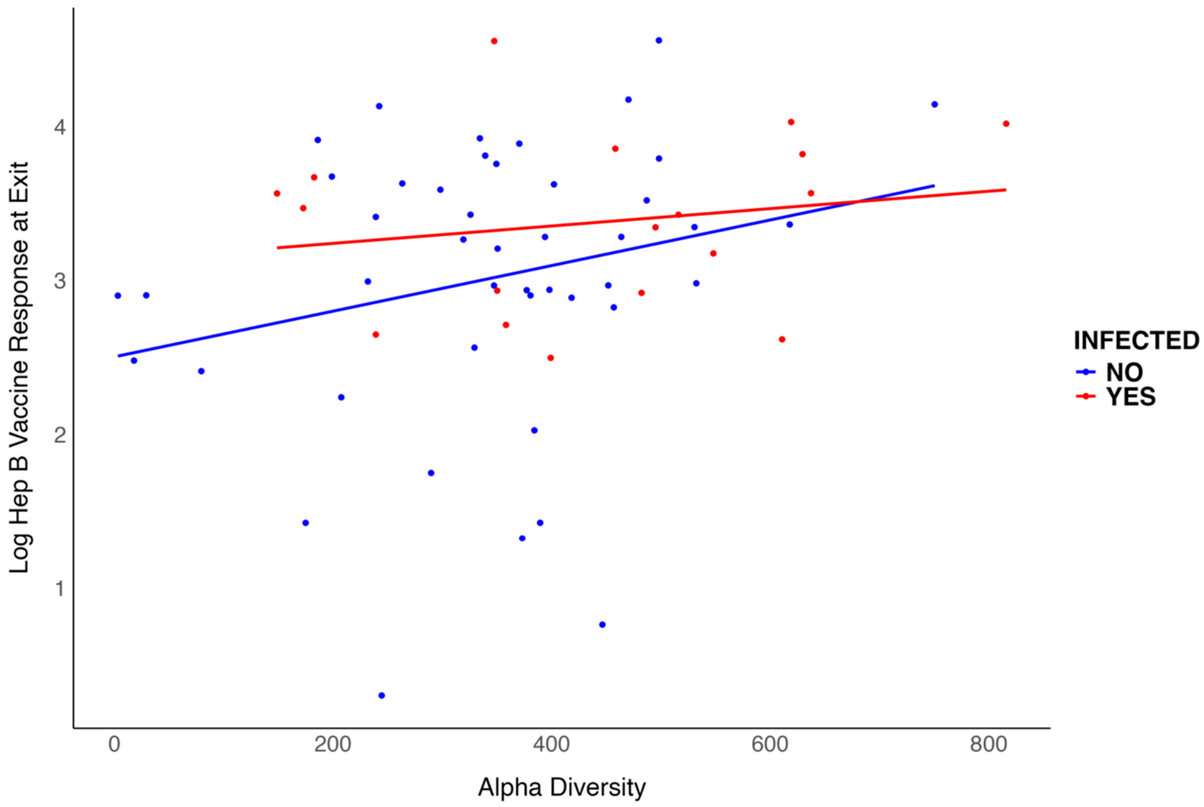

Log Hep B Vaccine Response at Exit
1
2
3
4
0
200
400
600
800
Alpha Diversity
INFECTED
NO
YES

S3. Inverse Simpson

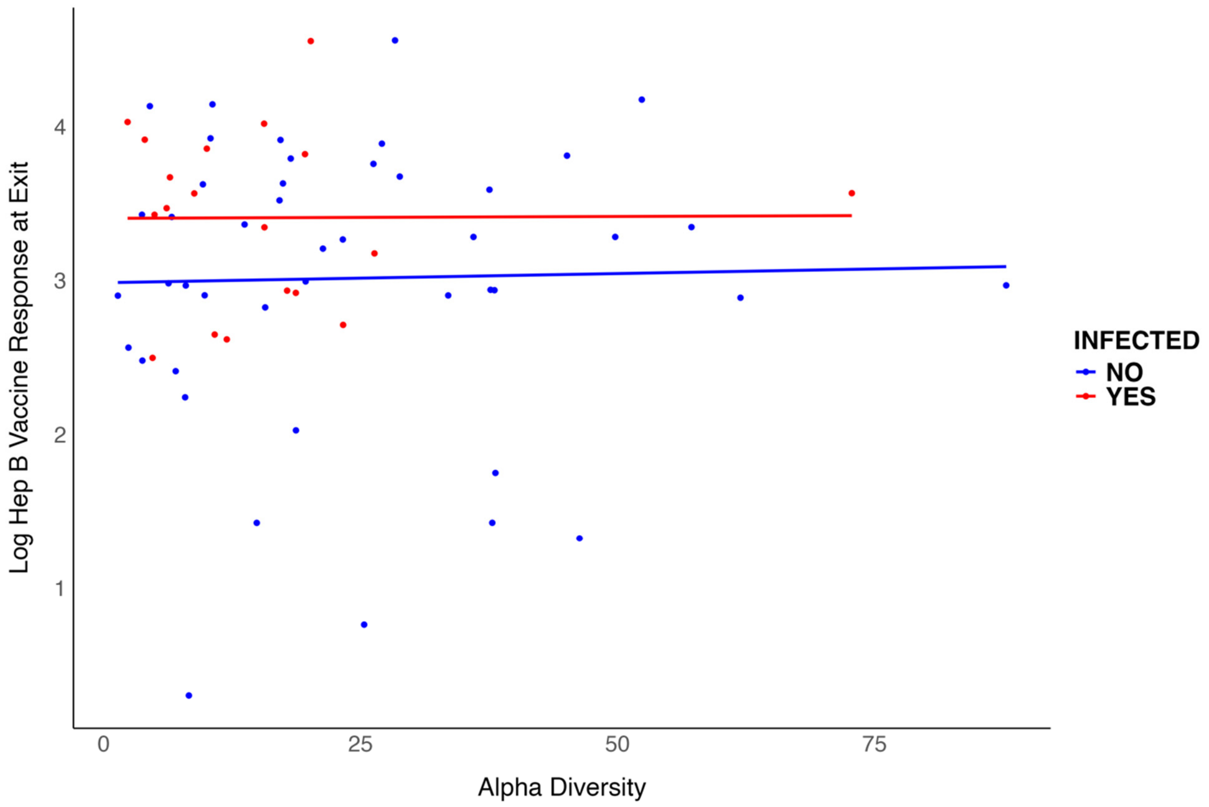

Supplement: Supplementary file 1 [file vaccines-13-00375-s001.zip › vaccines-3505154-supplementary.pdf]
